# Prevalence of Gastroesophageal Reflux Disease and Its Association with *Helicobacter pylori* Infection in Chronic Renal Failure Patients and in Renal Transplant Recipients

**DOI:** 10.4103/1319-3767.41741

**Published:** 2008-10

**Authors:** Ibraheim S. Abdulrahman, Abdulaziz A. Al-Quorain

**Affiliations:** Department of Internal Medicine, Nephrology Division, King Fahad University Hospital, Al-Khobar, Saudi Arabia; 1 Department of Internal Medicine, Gastroenterology Division, King Fahad University Hospital, Al-Khobar, Saudi Arabia

**Keywords:** Chronic renal failure, gastroesophageal reflux, *helicobacter pylori*

## Abstract

**Background/Aims:**

The prevalence of gastroesophageal reflux disease (GERD) in chronic renal failure patients and in renal transplant recipients (RTR) has been a subject of discussion in the last few years. Our aims are to clarify this association and its relation to *Helicobacter pylori* infection, and also to identify possible pathogenic factors in the development of this disease in both groups.

**Methods:**

The study involved 40 end-stage renal disease (ESRD) patients with upper gastrointestinal (GI) symptoms (group I), 36 patients who had undergone kidney transplantation and had similar symptoms (group II), and 44 age- and sex-matched controls with the same upper GI symptoms (group III). All patients were subjected to esophagogastroduodenoscopy, and biopsies were obtained from the antrum for histological evaluation and identification of *H. pylori*.

**Results:**

The prevalence of GERD in the first two groups was similar (77.5 vs. 75.0%, *P* = 0.412), while it was significantly lower in the control group (38.6%, *P* < 0.01). *H. pylori* infection was present in 40.0, 36.1 (*P* > 0.05) and 75% (*P* < 0.01 and < 0.001) of the patients in groups I, II, and III, respectively. Multivariate logistic regression analysis in groups I and II showed that high serum creatinine (Odds ratio [OR] = 6.78, 95% Confidence Interval (CI) = 1.12-45.82), immunosuppressive therapy (OR = 5.78, 95% CI = 1.01-32.5), and absence of *H. pylori* infection (OR = 3.58, 94% CI = 1.11-18.6) were significantly associated with GERD. The duration of ESRD correlated significantly with the prevalence of GERD in group I.

**Conclusions:**

This study showed a similar prevalence of *H. pylori* infection and GERD in ESRD and RTR patients. GERD prevalence was higher in these two groups than in the controls. Renal transplantation, chronic renal disease, immunosuppressive therapy, and the absence of *H. pylori* infection seem to be risk factors for the development of GERD.

***Helicobacter pylori*** infection is accepted as an etiological factor of chronic gastritis, peptic ulcer disease, and other gastrointestinal (GI) disorders.[1–4] Gastrointestinal symptoms, particularly heartburn, pyrosis, and regurgitation, are frequent findings in end-stage renal disease (ESRD) patients and in renal transplant recipients (RTRs). These complaints may be due to gastroesophageal reflux disease (GERD).[4,5] GERD describes the clinical manifestations of reflux of gastric contents into the esophagus. Although the exact prevalence is difficult to determine, it seems that GERD is the most common esophageal disease seen in primary care settings. The prevalence of GERD is now increasing; however, little is known about this condition in ESRD and RTRs. Although upper GI diseases and their complications are frequent in both patient groups, only few reports are available on the prevalence of ***H. pylori*** and its influence on dyspepsia and GERD in ESRD and RTRs. In a recent study from Germany, the prevalence of ***H. pylori*** was found to be significantly lower in chronic uremia patients than in nonuremics, probably due to the uremia which protects against ***H. pylori*** infection.[6] In other studies however, the ***H. pylori*** prevalence was found to be higher.[7,8] Kashiwagi ***et al.*** found that ***H. pylori***-positive subjects accounted for only 23.5% of the RTRs.[8] The aims of this study are to investigate the prevalence of GERD in symptomatic ESRD and RTR patients and its association with ***H. pylori*** infection.

## PATIENTS AND METHODS

End-stage renal disease patients, RTRs, and nonrenal patients with symptoms suggestive of GERD, were interviewed. A questionnaire was used to assess renal disease (etiology, duration, and type of treatment), symptoms of GERD, history of drug therapy, previous ***H. pylori*** eradication therapy, and concomitant chronic diseases. Patients who had taken antibiotics within 4 weeks of this study, those with other chronic illnesses, or taking drugs with potential GI motility effects, were excluded. A signed consent was obtained from every patient enrolled in this study. This prospective study was carried out according to the guidelines of the Medical Ethical Committee of King Faisal University in Dammam and the Helsinki Declaration. A total of 120 patients fulfilled the inclusion criteria and were grouped as follows: group I = 40 (33.3%) ESRD patients; group II = 36 (30%) RTRs, and group III = 44 (36.7%) dyspeptic patients without renal disease. Mean ages, male/female ratios, and other patient characteristics are shown in Table 1. The mean chronic renal disease duration for group I was 39 ± 18.6 months and the mean posttransplantation time for group II was 36.26 ± 15.8 months. All ESRD patients were on calcium supplementation, IV calcitriol, folic acid, and erythropoietin therapy. All RTRs used CellCept^®^, cyclosporine-A, and prednisolone as immunosuppressive therapy; serum blood levels were monitored to modify the dosages. All patients were subjected to upper GI endoscopy with Olympus GIF Q 230 videofibroscope; GERD was diagnosed endoscopically according to the Los Angeles (LA) classification. Biopsies were obtained from the lower esophagus, the gastric antrum and the body, and from ulcer edges (if present) for histological evaluation and the presence of ***H. pylori***. All biopsy specimens were interpreted by a single histopathologist.

**Table 1:** Demographic characteristics of patients

	Group I (ESRD)	Group II (RTR)	Group III (Non-renal)	*P* value
Patients (*n*)	40	36	44	NS**
Mean age (years)	46.4 ± 15.7	51.8 ± 12.0	48.6 ± 12.3	NS
Male/female (*n*)	24/16	21/15	26/18	NS
Mean duration of ESRD (months)	39 ± 18.6	-	-	
Mean post transplant duration	-	36.26 ± 15.8	-	
Serum creatinine mg/dL (mean)	12 ± 4.4	1.6 ± 1.1	0.9 ± 0.3	< 0.001^ab^
				< 0.001^ac^
				NS^bc^**

*NS- Not significant

** ab- group I vs. group II, ac- group I vs. group III, bc- group II vs. group III

Statistical analyses of the recorded data were performed using standard commercially available computer software (Synetics Medical). Numerical values were given as mean ± standard deviation (SD) and t-test was used when appropriate. For nominal data, the χ^2^ -test was utilized. Fisher exact test was applied, and ***P*** values < 0.05 were accepted as being significant. Multivariate logistic regression analysis was used with stepwise backward variable selection to test for factors that predicted the presence of GERD. Results were considered significant when they were beyond the 0.05 level of probability. The ORs from logistic regression were also presented as measures of the strength of associations.

## RESULTS

The three groups were well matched in terms of their mean ages and male/female ratios (***P*** > 0.05) [Table 1]. Thirty-one (77.5%) patients in group I, 27 (75.0%) in group II, and 17 (38.6%) in group III showed endoscopic and histopathologic findings of GERD. The differences in the findings between group I patients and those of groups III and II (***P*** < 0.01) were of statistical significance. Sixteen (40.0%) patients in group I, 13 (36.1%) in group II, and 33 (75%) in group III with nonulcer dyspepsia (NUD), were positive for ***H. pylori***. The difference in the prevalence rates between the first two groups was statistically insignificant (***P*** > 0.05), whereas the prevalence of ***H. pylori*** infection in group III was significantly higher than the other two groups (***P*** < 0.01 and ***P*** < 0.001, respectively). Multivariate logistic regression analysis of groups I and II showed that high serum creatinine (>7.0 mg/dl) (Odds ratio [OR] = 6.78, 95% Confidence Interval [CI] = 1.12-45.82), immunosuppressive therapy (OR = 5.78, 95% CI = 1.01-32.5), and absence of ***H. pylori*** infection (OR = 3.58, 95% CI = 1.11-16.8) were significantly associated with GERD. The endoscopic findings and ***H. pylori*** status of the groups are shown in Tables 2 and 3. Positive correlation was observed between the prevalence of GERD and the duration of ESRD but not with the duration of elapsed period after the renal transplant (***P*** < 0.01 and ***P*** > 0.05, respectively) [Table 4].

**Table 2:** Endoscopic findings in each group

	Group I (ESRD)		Group II (RTR)		Group III (NR*)	
			
	*n*:40	(%)	*n*:36	(%)	*n*:44	(%)
Gastritis	14	35	10	27.8	9	20.5
Gastric ulcer	3	7.5	-	-	1	2.3
Duodenal ulcer	5	12.5	5	13.9	9	20.5
GERD	31	77.5	27	75	17	38.6
Hiatus hernia	1	2.5	-	-	-	-

* NR- Nonrenal, ESRD- End-stage renal disease, RTR- Renal transplant recipients

**Table 3:** *H. pylori* prevalence in each group

	*H. pylori* (+) (%)	*H. pylori* (-) (%)	Total
Group I (ESRD)	16 (40)	24 (60)	40
Group II (RTR)	13 (36.1)	23 (63.9)	36
Group III (NR*)	33 (75.0)	11 (25.0)	44

* NR- Nonrenal, ESRD- End-stage renal disease, RTR- Renal transplant recipients, *H. pylori: Helicobacter pylori*

**Table 4:** Relationship of GERD prevalence with duration of ESRD and with duration of time elapsed posttransplantation

	GERD (+)	GERD (-)	*P*
Duration of ESRD (months)	47.85 ± 19.22	26.63 ± 13.45	<0.01
Duration of postrenal transplant (months)	39.53 ± 20.47	31.14 ± 17.38	>0.05

*NR- Nonrenal, GERD- Gastroesophageal reflux disease, ESRD- End-stage renal disease

## DISCUSSION

The pathophysiology of GERD is multifactorial and depends on the interaction between defense mechanisms and pathophysiological factors. The lower esophageal sphincter (LES) normally works in conjunction with the diaphragm to create a physical barrier against the reflux of gastric contents into the esophagus.[9] Transient relaxation of the LES may occur more often in patients with ESRD.[10] Esophageal motility disorders and delayed gastric emptying may also play a role in the development of GERD in ESRD patients.[11]

The role of delayed gastric emptying remains controversial, but patients with gastroparesis such as chronic renal failure patients, have shown a predisposition to gastroesophageal reflux.[12] In a recent publication, Strid and his colleagues have found delayed gastric emptying in 36% of their patients with ESRD, with a higher prevalence in peritoneal dialysis patients compared to chronic renal failure patients who are not on dialysis. This might be explained by the increased intra-abdominal pressure induced by the intraperitoneal dialysis fluid along with other factors.[13] Findings have been inconsistent regarding the relationship between gastric acid secretion and uremia; however, most authors believe that chronic renal failure is associated with hyperchlorhydria.[14–17] Moreover, individuals with impaired renal function have elevated serum gastrin levels due to reduced excretion and impaired metabolism in the liver degradation of gastrin.[14,16–19] Straathof ***et al.***[20] reported that postprandial plasma concentrations of gastrin, in addition to its effect on gastric acid secretion, decrease LES pressure and increase the transient LES relaxations associated with reflux. These factors collectively may explain the higher prevalence of GERD in our group of patients with ESRD.

Multifactorial logistic regression analysis of various factors in relation to GERD in our ESRD patients identified two independent parameters which were significantly associated with reflux esophagitis, namely, high serum creatinine levels and the absence of ***H. pylori*** infection [Table 5].

**Table 5:** Factors that predict GERD based on endoscopic and microscopic esophagitis findings (Multivariate logistic regression analysis results)

	*P*	OR	95% CI
High serum creatinine (> 7 mg/dL)	0.0243	6.7836	1.12-45.82
*H. pylori* (-)	0.0417	3.58	1.11-16.8
Immunosuppressive therapy	0.0219	5.7819	1.01-32.5

*H. pylori- Helicobacter pylori*, GERD- Gastroesophageal reflux disease

The role of ***H. pylori*** in GERD is highly controversial.[21] It is well known that ***H. pylori*** infection of the antrum does not affect gastric acid secretion, while ***H. pylori*** chronic corpus gastritis causes hypochlorhydria due to a decrease in the number of the parietal cells.[22] El-Serag ***et al.***[23] reported that corpus gastritis is protective against reflux esophagitis. Consistent with this finding, all our ***H. pylori***-positive ESRD patients had corpus gastritis, resulting in hypochlorhydria. This could explain our finding of a negative relationship between ***H. pylori*** gastritis and GERD. Thus, it could be argued that ***H. pylori*** infection protects against the development of GERD by decreasing acid secretion and also by affecting mucosal defense mechanisms in the esophagus.

In our study, the prevalence of ***H. pylori*** gastritis was lower in ESRD patients than in the controls (***P*** < 0.01) [Table 3]. This may have translated into a relatively higher GERD prevalence in the ESRD group compared to the controls (77.5 vs. 38.6%, ***P*** < 0.01). The frequency of GI problems increases after renal transplantation.[24] In a study from Spain, 480 RTRs were observed to have 79 GI complications of which GERD, esophagitis, and chronic gastritis were among the most frequently observed ones. Upper GI bleeding occurred in 60% of them, which is a much higher incidence than that observed in the general population.[25]

One might expect a high ***H. pylori*** prevalence among RTRs secondary to immunosuppressive therapy, but this was not the case in our study which showed a prevalence of 36.1 vs. 75% in the controls (***P*** < 0.001) [Table 3]. Teenan ***et al.*** and Davenport ***et al.*** reported a prevalence of 48 and 29%, respectively.[26,27] In the study by Teenan ***et al.***, no relationship was observed between ***H. pylori*** colonization and cyclosporine or prednisolone levels,[26] suggesting other possible mechanisms for the lower prevalence of ***H. pylori*** and hence, the higher frequencies of GERD in this group of patients. Possible protective mechanisms include the frequent use of antibiotics in RTRs[28] resulting in changes in bacterial colonization of the upper GI tract.[29] In addition, an alteration in the protective balance due to the use of antacids, histamine-2 receptor antagonists, proton pump inhibitors (PPIs), and stasis of the GI tract can lead to inhibition of ***H. pylori*** growth and overgrowth of pathogenic and resistant gram-negative and gram-positive bacteria and fungi.[30]

## CONCLUSION

This study showed a similar prevalence of ***H. pylori*** infection and GERD in ESRD patients and RTRs. The results of this study support the recommendation that all ESRD patients with upper GI symptoms should be evaluated for GERD. Renal transplant recipients seem to be at increased risk of GERD due to multiple factors. The role of immunosuppressive therapy in this context is not yet clear. ***H. pylori*** infection of the corpus might protect against GERD in both ESRD and RTR patients. This study represents one of the largest cohorts of ESRD and RTR. A larger cohort of patients is needed to further address possible mechanisms of postrenal transplant reflux disease, including the effects of specific immunosuppressive medications.
